# Activity-Based Screening of Soil Samples from Nyingchi, Tibet, for Amylase-Producing Bacteria and Other Multifunctional Enzyme Capacities

**DOI:** 10.1155/2022/2401766

**Published:** 2022-11-21

**Authors:** J. H. Liu, J. N. Guo, H. Lu, J. Lin

**Affiliations:** Shanghai Engineering Research Center of Industrial Microorganisms, State Key Laboratory of Genetic Engineering, School of Life Sciences, Tibet University-Fudan University Joint Laboratory for Biodiversity and Global Change,Fudan University, Shanghai 200438, China

## Abstract

Despite the interest in Tibetan soil as a promising source of functional enzymes with potential biotechnological applications, few studies have considered the screening and identification of amylase producing bacteria from Tibetan soil. Amylase has many applications in the food and feed industries, textile and biofuel production, and biomedical engineering. The area of amylase with specific properties is attracting growing attention because of its better application to various industrial conditions. This study aims to screen and identify amylase-producing strains from soil samples collected in Nyingchi, Tibet, and then explore whether the bacterial isolates are superior for unique enzymes. In this paper, a total of 127 amylase producing bacteria were isolated by activity-based screening of six Tibetan soil samples. The 16S rRNA gene survey then identified four major phyla, namely, firmicutes, bacteroidetes, proteobacteria, and actinobacteria, which were differentiated into twelve genera with a dominance of *Bacillus* (67.72%), followed by *Pseudomonas* (8.66%). Microbial diversity analysis revealed that the amylase-producing bacterial community of the Kadinggou forest soil sample showed the best variety (the Simpson index was 0.69 and the Shannon index was 0.85). The amylase activity assay of the bacterial isolates showed a mean of 0.66 U/mL at 28°C and pH 5.2. Based on the effect of temperatures and pHs on amylase activity, several bacterial isolates can produce thermophilic (50°C), psychrophilic (10°C), acidophilic (pH 4.2), and alkaliphilic (pH 10.2) amylases. Furthermore, four bacterial isolates were screened for amylase, protease, and esterase activities, which indicated multifunctional enzyme capacities. The present study is expected to contribute to our understanding of Tibetan microbial resources and their potential for scientific research and industrial applications.

## 1. Introduction

As the most active members of the terrestrial ecosystem, soil microorganisms participate in various biochemical processes such as the decomposition of organic matter, the transformation of nutrients, and the formation of humus in the soil environment and play a vital role in the regulation of material circulation and the maintenance of ecological stability. There is a wide variety of microorganisms in the soil, which is considered an essential resource for obtaining many microbial enzymes. Amylase, 1, 4-*α*-D-glucanohydrolase, can catalyze the hydrolysis of starch, glycogen, and related polysaccharides into glucose, maltose, and other oligosaccharides. Due to one of the most critical hydrolases in industrial starch production, amylase was commercially applied in 1894 as a medical adjuvant for treating digestive disorders. Presently, amylase has become the most widely used enzyme preparation with the most significant yield, accounting for 30 percent of the world's enzyme market [1, 2]. Amylases have extensive applications in the food and feed industry, textile and biofuel production, paper and pulp technology, biopharmaceutical engineering, etc. [2–4]. Microbial fermentation has thus far been the primary method for large-scale amylase production. Previous studies have reported that amylase-producing bacteria belong mainly to the genus *Bacillus* for industrial applications, such as *B. subtilis*, *B. licheniformis*, *B. amyloliquefaciens, B. coagulans,* and *B. stearothermophilus* [3–5]. In addition, commercially available fungal amylases are produced by the genus *Aspergillus* (*A. oryzae* and *A. niger*), and the *Penicillium* species is also an efficient amylase-producing fungus [5, 6].

It should be noted that with the development of the starch processing industry and the expansion of amylase applications, the investigation of amylase resources with superior characteristics has been considered a hot issue in the current enzyme preparation industry. For example, high-temperature amylase is thermostable within the temperature range of 70–90°C, which can be of vital industrial interest in starch processing for energy conservation and economic benefits [7, 8]. The most thermostable *α*-amylases used in industry are produced from *B. licheniformis*, BLA [9–11]. Besides, a hyperthermophilic extracellular *α*-amylase from *Pyrococcus furiosus* (PFA) had its optimal activity at 100°C, which was significantly more thermostable than the commercially available BLA [12]. Furthermore, *B. subtilis* and *B. amyloliquefaciens* are responsible for producing medium-temperature (30–40°C) amylases [3, 8] that are applied to eliminate the complicated steps of the removal of calcium ions in the starch process [10]. While, low-temperature amylase is generally characterized by a specific enzyme activity at low temperatures, exemplified by the well-known psychrophilic *Alteromonas haloplanctis α*-amylase (AHA) showing its specific activity within a temperature range of 0–20°C [13, 14]. The cold-active property confers to psychrophilic amylase a tremendous economic value and a broad application prospect, as the mesophilic amylases have their activity greatly reduced under low temperatures.

In addition to the above-mentioned temperature features (thermophilic, mesophilic, and psychrophilic), other functional traits of amylases adapted to a particular environment are also supposed to be significant for various industrial applications. For example, an acid-stable amylase from *Bacillus* sp. KR-8104 [15], which maintains its specific activity at low pH values (pH 4.0–6.0), is more favorable for the liquefaction and saccharification processes due to the omission of pH adjustment steps and the limitation of byproducts formation [11]. Furthermore, halophilic [16], organic solvent tolerant [17], detergent compatible [18], and metal ion independent microbial amylases [19] can also meet the requirements of current industrial applications. Concerning product output improvement and total cost reduction, amylases with multiple properties can attract more attention in industrial starch processing, such as a thermostable and acid stable amylase from *B. licheniformis* B4-423 [11], a thermophilic and calcium independent amylase from *Bacillus* sp. TM1 [19], a halophilic alkali and thermostable amylase from *Amphibacillus* sp. NM-Ra2 [20], and a mesophilic and acidophilic amylase from *Lactobacillus plantarum* subsp. *plantarum* ST-III [21]. Collectively, the amylases bearing superior properties can withstand the harsh conditions of industrial processing without denaturation, which allows them to be used as efficient biocatalysts in diverse reaction conditions.

Even though the world's amylase industry has developed significantly, it is difficult for our country, China, to have its place on the international market due to fewer varieties and yields of industrialized amylase. Therefore, it is urgent for us to vigorously develop the domestic industry. On the one hand, the exploration of new amylase-producing microbial resources remains a crucial goal. On the other hand, further screening of amylases for specific adaptive characteristics (e.g., thermophilic, psychrophilic, acidophilic, and alkaliphilic amylases) in a unique natural environment should be focused mainly on [22, 23]. To date, microbial amylases with unique properties isolated from water samples (deep sea, hot springs, and lakes) have been well documented [11, 20, 24–26]. In contrast, soil-derived amylase-producing bacteria with superior enzymatic characteristics have received relatively little attention.

Nyingchi city, located in the southeast of Tibet, is a vital forest resource area and a prominent grain product region. According to the data from Weather China (http://www.weather.com.cn), the city is characterized by annual mean temperature of 8.7°C, annual sunshine time of 2,000 hours, an annual precipitation of 650 millimeters, and an average altitude of 3,100 meters. A recent study reported that mean annual precipitation and mean annual temperature significantly influenced the structure of the soil microbial community [27]. Additionally, diverse climatic types (tropical, subtropical, temperate, frigid, humid, and subhumid zones) confer to Nyingchi a greater variety in soil microbial ecosystem compared with other cities. Collectively, we believe that Tibetan soil, likely inhabited by specialized microbes or extremophiles, is an exciting source for searching for unique microbial amylases. Jiang et al. [28] in 2007 reported a bacterial isolate (*Bacillus licheniformis* LT) with thermostable *α*-amylase from Tibetan hot springs. Quan et al. [29] in 2015 preliminarily studied the distribution of amylase-producing bacterial isolates from soil samples collected on the Qinghai-Tibetan Plateau.

In the present study, our main objective was to (i) screen amylase producing bacteria from soil samples collected in Nyingchi city, Tibet, using an activity-based approach; (ii) illustrate microbial taxonomy, composition, and diversity among different soil samples using the 16S rRNA gene sequence survey method and the calculation of various diversity indices; (iii) obtain unique bacterial isolates for producing superior amylases through the study on the optimal temperature and pH of enzyme activity; (iv) and explore the multifunctional enzyme capacities of our bacterial isolates. Our study hopes to realize the transformation from simple discovery to high value utilization of Tibetan microbial amylase resources.

## 2. Materials and Methods

### 2.1. Study Site and Sample Collection

**Figure 1:**
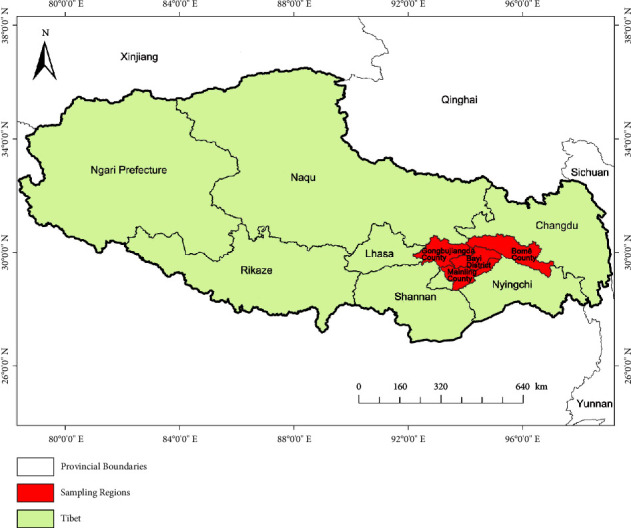
The map of sampling sites.

The soil samples in this study were collected from four sites in Nyingchi city, Tibet (i.e., Guomo village, Mainling County, Runa village, Bomê County, Basongcuo, and Kadinggou). The samples were divided into two types: crop soil and forest soil. The crop soil was subdivided into buckwheat, highland barley, and corn. Together, six soil samples were named GM-b, GM-hb, RB-c, RB-hb, BSC-f, and KDG-f. The geographic locations of the sampling are shown in Figure 1. Detailed information on all samples is described in Table 1.

2 cm of topsoil was removed during sample collection. The mixed sample of 2–15 cm from the ground was collected into sterile containers and then transported to the laboratory, where it was temporarily stored at 4°C for subsequent experiments.

### 2.2. Culture Medium and Chemicals

Tryptic soy agar medium (TSA) and tryptic soy broth (TSB) were purchased from Hope Bio Technology (Qingdao, China). The formula and directions can be found on the reagent bottles. Glyceryl tributyrate, 3, 5-dinitrosalicylic acid (DNS) reagent, and sodium acetate buffer were obtained from Solarbio (Beijing, China). Soluble starch, D-glucose, iodine, potassium iodide, nonfat powdered milk, and all other chemicals were provided by Sangon Biotech (Shanghai, China) and were of grade BC.

### 2.3. Isolation and Screening of Amylase-Producing Strains

2 g of soil samples were added to 22.5 mL of sterile water in an Erlenmeyer flask. Mix the sample vigorously with a vortex. After holding the deposition for 15–20 minutes, 1 mL of suspension was added to 9 mL of sterile distilled water, and a serial dilution (10^−1^ to 10^−3^) was prepared. About 100 *µ*l of each dilution was added and distributed on amylase-screening TSA plates [30] containing 0.2% (w/v) soluble starch. The plates were incubated at 28°C for 16–18 hours until typical bacterial colonies were observed. Starch hydrolysis was examined by fumigating the plates with iodine for 1 minute [31], and positive bacterial colonies with amylase activity were selected through visible hydrolytic circles against a dark blue background. The colonies showing a clear difference in morphology were further purified by subculture on amylase-screening TSA plates until a monoclonal cell of uniform morphology and size was observed [32]. The pure isolates were transferred to TSB and incubated at 28°C at 220 rpm for 16–18 hours.

### 2.4. Identification and Classification of Amylase-Producing Strains

16S rDNA analysis was performed to identify species and classify the taxonomy of bacterial isolates. 16S bacterial rDNA was amplified using universal primers 27-F (5′-AGAGTTTGATCCTGGCTCAG-3′) and 1492-R (5′-TACGGTTACCTTGTTACGACTT-3′) [11]. PCRs were run at a volume of 50 *µ*l using the Phanta® Max Master Mix (Vazyme, Nanjing, China) which can be used for direct PCR amplification of bacterial samples without extraction of genomic DNA. The PCR system contained (*µ*l): 2 × Phanta Max Master Mix 25.0, bacterial suspension as template 1.0, 27-F (10 *µ*M) 2.0, 1492-R (10 *µ*M) 2.0, and ddH_2_O 20.0. The PCR program consisted of initial denaturation at 95°C for 3 minutes, followed by 30 cycles of denaturation at 95°C for 15 seconds, annealing at 55°C for 15 seconds, and elongation at 72°C for 90 seconds, with a final extension time of 5 minutes at 72°C. After the 1.5 kb target fragments of the PCR amplicons were verified with 1% agarose (Biowest, Spain) gel electrophoresis, the PCR products were sequenced from Sanger sequencing technology at Tsingke Biotechnology Co., Ltd. (Beijing, China). Subsequently, the bacterial 16S rDNA sequences were submitted into two online public databases, NCBI Genebank (https://blast.ncbi.nlm.nih.gov/Blast.cgi) and EZBioCloud (https://www.ezbiocloud.net/identify), to identify the nearest previously described bacteria and to locate the top-hit taxonomy [33]. Compare and verify the identification results of the two databases to determine the classification of the bacteria isolates.

### 2.5. Calculation of Bacterial Community Diversity Indices

The microbial diversity was assessed by calculating and comparing ecological indices including the Berger–Parker index (*D*_*B-P*_) of dominance, the Margalef index (*d*_*Ma*_) of richness, Simpson index (*D*) of inhomogeneous probability, the Shannon–Wiener index (*H′*) of diversity, and the Pielou index (*J*_*e*_) of evenness. These parameters were calculated according to equations (1)–(5) [34–48]:

(1) $$\begin{array}{c} {Berger-Parker\;in\;de\;x,D}_{B-P}=\frac{n_{\max}}{N}\text{,} \end{array}$$

(2) $$\begin{array}{c} {Margalef\;in\;de\;x,d}_{Ma}=\frac{S-1}{InN}\text{,} \end{array}$$

(3) $$\begin{array}{c} Simpson\;in\;de\;xD=1-\overset{S}{\underset{i=1}{\sum }}{\left(P_{i}\right)}^{2},P_{i}=\frac{n_{i}}{N}\text{,} \end{array}$$

(4) $$\begin{array}{c} {Shannon–Wiener\;in\;de\;x,H}^{\prime }=-\overset{S}{\underset{i=1}{\sum }}P_{i}InP_{i}\text{,} \end{array}$$

(5) $$\begin{array}{c} {Pielou\;in\;de\;x,J}_{e}=\frac{H^{\prime }}{H_{\max}^{\prime }}=\frac{H^{\prime }}{InS}\text{.} \end{array}$$

*n*_max_ is the number of the most appeared species, *N* is the sum of the individual numbers of all species, *S* is the number of species, *P*_*i*_ is the proportion of individuals belonging to *i*th species, and *n*_*i*_ is the number of the *i*th species.

*D*_*B-P*_ is the dominance of the most abundant species, which indicates that a sample with a higher species diversity corresponds to a lower *D*_*B-P*_ value. While *D* represents the probability of two randomly sampled individuals belonging to different species (i.e., inhomogeneous probability), a sample with a higher species diversity corresponds to a higher *D* value. As an index of quantifying entropy (uncertainty) and characterizing diversity, *H′* is proportional to both the Margalef index of richness and the Pielou index of evenness. There are two critical components of community diversity: *d*_*Ma*_ emphasizes species richness with intuitive ecological significance, and *J*_*e*_ reflects the distribution of all individuals in a community with sensitivity to species richness [41, 42].

### 2.6. Determination of Amylase Activity

The amylase-producing bacteria isolates were incubated in TSB medium for 16–18 hours. The culture medium was centrifuged at 12000 rpm for 20 minutes, and the supernatant was collected as the crude enzyme solution for a subsequent enzyme activity assay. Amylase activity was determined using the Bernfeld method [39], using 3, 5-dinitrosalicylic acid (DNS) to measure the reducing sugars released during amylolysis. The 0.8 mL reaction mixture was composed of 20 mM sodium acetate buffer (pH 5.2; 0.36 mL); 1.0% (w/v) soluble starch dissolved in the buffer (0.4 mL); and a crude enzyme solution (0.04 mL). After incubation at 28°C for 15 minutes, the DNS reagent was boiled for 10 minutes to develop color and then cooled to room temperature. The release of reducing sugars was determined spectrophotometrically at 540 nm [32, 40]. One unit of amylase in our assay was defined as the amount of enzyme that liberated 1.0 *µ*mol of reducing sugar per minute in 1.0 mL of crude enzyme solution under certain assay conditions. Equation (6) of the amylase activity was calculated as follows:

(6) $$\begin{array}{c} relative\;amylase\;activity=\frac{A\times V_{1}\times 10^{3}}{t\times V_{2}\times 180}\left(\frac{\mu mol}{ml∙\min}\right)\text{.} \end{array}$$

*A* was the concentration of reducing sugars (mg/mL), *V*_1_ was the total reaction volume (mL), *t* was the reaction time (minute), and *V*_2_ was the crude enzyme volume (mL).

### 2.7. The Study of Enzyme Properties in Amylase-Producing Strains

#### 2.7.1. Effect of Temperatures and pHs on Amylase Activity

The crude enzyme source was further characterized for the amylase activity under different temperatures (4, 10, 28, 50, and 65°C) and pH values (pH 3.2, 4.2, 5.2, 9.2, and 10.2) to determine the optimum reaction condition.

#### 2.7.2. Screening of Bacterial Isolates for Multifunctional Enzyme Capacities

2 *µ*l of bacterial culture medium was added to protease-screening TSA plates supplemented with nonfat powdered milk (1.0%, w/v) [41] and esterase-screening TSA plates containing 1.0% (w/v) glyceryl tributyrate [42]. Hydrolysis was directly examined by the clear zones around the growth margin.

## 3. Statistical Analysis

The amylase activity assay was determined with three independent replicates, and the data were the mean of three parallel experiments. Statistical analysis for significant differences between mean values was performed with a one-way ANOVA at the 95% level (*p* value <0.05) using the software GraphPad Prism (version 9).

## 4. Results and Discussions

### 4.1. Screening of Amylase-Producing Bacteria from Tibetan Soil

By the activity-based screening method, we obtained 127 strains with amylase activity among 1,184 bacterial colonies from six soil samples, accounting for 10.73%. The statistical results indicated that the sample KDG-f possessed the highest isolation proportion of amylase-producing strains, reaching 17.50%, followed by GM-b (15.52%). The detailed screening results of the six samples are described in Table 2. In terms of crop soil types, buckwheat had a better isolation ratio (GM-b: 15.52%) than corn and highland barley (RB-c: 9.68%, GM-hb: 8.22%, and RB-hb: 7.51%). Therefore, we raise the hypothesis that bacterial isolates for amylase may be associated with crop soil types, which needs to be further investigated.

**Figure 2:**
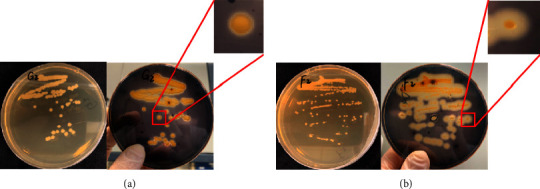
Two types of amylase-producing bacterial colonies. (a) A bigger colony with a smaller zone of hydrolysis, and (b) a smaller colony with a bigger zone of hydrolysis.

We obtained two types of amylase producing bacterial colonies during the obtaining of pure bacterial isolates: a bigger colony with a smaller zone of hydrolysis (Figure 2(a)) and a smaller colony with a bigger zone of hydrolysis (Figure 2(b)). Then, we preliminarily speculated that the latter has a better ability to produce amylase based on the diameter ratio of the hydrolytic circle to the bacterial colony.

### 4.2. Identification and Classification of Amylase Producing Bacteria

**Figure 3:**
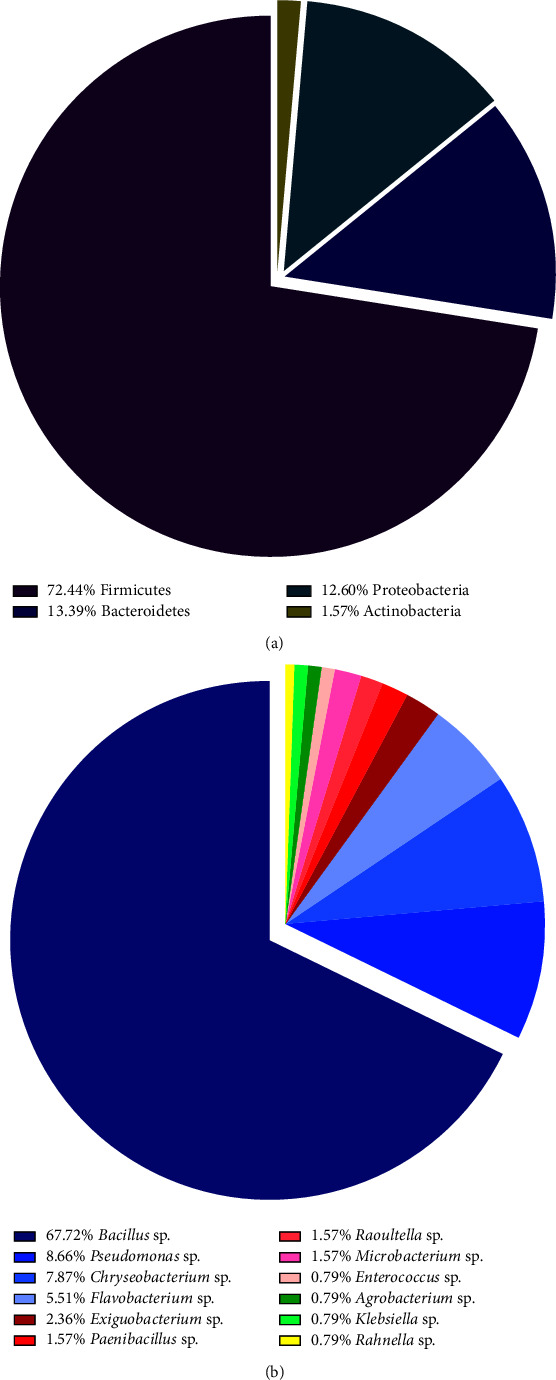
Taxonomic classification of the 127 amylase-producing bacteria on (a) bacterial phylum level and (b) bacterial genus level.

Subsequently, amylase-producing bacterial isolates were identified and classified. From a phylogenetic analysis of publicly available 16S rRNA gene sequences, 127 strains belonged to four phyla (Figure 3(a)), that is, firmicutes (72.44%), bacteroidetes (13.39%), proteobacteria (12.60%), and actinobacteria (1.57%). As can be seen in Figure 3(b), the taxonomy of the bacterial genera revealed a dominance of *Bacillus* sp. (a total of 86 strains, accounting for 67.72%), followed by *Pseudomonas* sp. (11 strains, 8.66%), *Chryseobacterium* sp. (10 strains, 7.87%), *Flavobacterium* sp. (7 strains, 5.51%), *Exiguobacterium* sp. (3 strains, 2.36%), *Paenibacillus* sp. (2 strains, 1.57%), *Raoultella* sp. (2 strains, 1.57%), *Microbacterium* sp. (2 strains, 1.57%), *Enterococcus* sp. (1 strain, 0.79%), *Agrobacterium* sp. (1 strain, 0.79%), *Klebsiella* sp. (1 strain, 0.79%), and *Rahnella* sp. (1 strain, 0.79%).

The 16S rRNA gene survey was applied to gain insight into the species variety associated with microbial amylase. Our result was in agreement with previous studies [3, 5, 43] in which *Bacillus* was the most common bacterial genus isolated for amylase secretion. In our investigation, *Bacillus* isolates were differentiated into nine bacterial species, among which *B. cereus*, *B. megaterium*, *B. mycoides*, *B. thuringiensis*, *B. aryabhattai*, and *B. wiedmannii* have been well documented for their amylase production [44–47]. However, *B. toyonensis*, *B. anthracis*, and *B. frigoritolerans* remain little reported. Additionally, we found that the ability to produce amylase also appeared in other bacterial species, such as *Klebsiella aerogenes, Exiguobacterium acetylicum, Paenibacillus xylanexedens,* and *Enterococcus faecalis*, which was supported by previous studies [48–50]. Nevertheless, three bacterial species we isolated for amylase activity, namely *Pseudomonas kribbensis*, *P*. *glycinae*, and *Microbacterium phyllosphaerae*, have never been reported or published in NCBI GeneBank for their coding genes for the corresponding amylases. Therefore, further research is needed to determine whether the bacterial species mentioned above are novel sources for microbial amylase.

According to the BLAST search against known homologous 16S rDNA sequences, 17 redundant strains can be omitted due to the same identity as the other existing isolates in our study, given in Table 3. For example, bacterial isolates 1D2 and 1D21 had a sequence similarity of 100.00% aligned with the closest representative bacteria, *Bacillus mycoides* BF1-5. While isolates 2A6, 2G4, and 2D5 showed identical sequence similarity (99.93%) with *Pseudomonas fluorescens *Pf0-1. Based on the 16S rDNA sequence similarity, a total of 110 isolates were identified as inhomogeneous individuals at the bacterial strain level. They were selected as the tested strains for our subsequent amylase activity assay.

### 4.3. Bacterial Composition and Diversity Analysis

**Figure 4:**
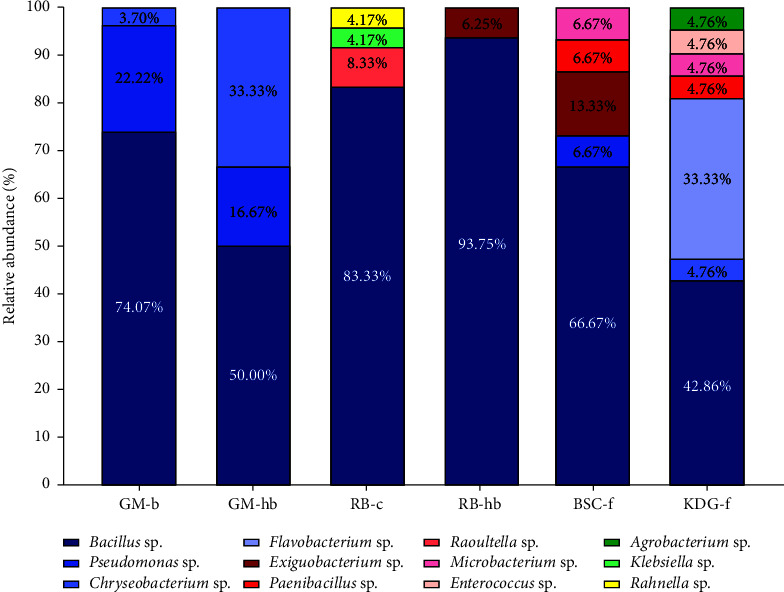
Taxonomy bar plot of the relative bacterial abundance of six Tibetan soil samples at the bacterial genus level.

More studies were required to determine how amylase-producing bacterial isolates were distributed among the different Tibetan soil samples. First, the taxonomy bar plot of relative abundance showed the bacterial composition and distribution of six soil samples (Figure 4). We can find that *Bacillus*, with the highest abundance, was supposed to be the dominant bacterial genus in six samples. Specifically, the relative abundance of *Bacillus* among six samples in order was RB-hb (93.75%), RB-c (83.33%), GM-b (74.07%), BSC-f (66.67%), GM-hb (50.00%), and KDG-f (42.86%). Furthermore, the sample KDG-f had the most bacterial genera (7 genera), followed by BSC-f (8 genera), RB-c (4 genera), GM-b (3 genera), GM-hb (3 genera), and RB-hb (2 genera). Figure 4 showed the details of the bacterial composition of six samples.

Secondly, the bacterial diversity of different samples was further assessed by the calculation of various diversity indices, as shown in Table 4. The sample RB-hb, with the highest dominance index (*D*_*B-P*_ = 0.94), the lowest richness index (*d*_*Ma*_ = 0.36), and the lowest evenness index (*J*_*e*_ = 0.09), was characterized by the worst bacterial diversity. The bacterial community was composed of two genera, with an overwhelming dominance of *Bacillus* (93.75%) over *Exiguobacterium* (6.25%). Additionally, the lowest Simpson index (*D* = 0.12) and Shannon–Wiener index (*H'* = 0.06) further revealed RB-hb as having the lowest bacterial diversity. However, the sample KDG-f with the lowest dominance index (*D*_*B-P*_ = 0.43) demonstrated the best bacterial diversity, which can be also proven by the highest Margalef index (*d*_*Ma*_ = 1.97), Simpson index (*D* = 0.69), and Shannon–Wiener index (*H'* = 0.85). The bacterial community consisted of seven genera, namely, *Bacillus* (42.86%), *Flavobacterium* (33.33%), *Chryseobacterium* (4.76%), *Paenibacillus* (4.76%), *Microbacterium* (4.76%), *Enterococcus* (4.76%), and *Agrobacterium* (4.76%). Regarding the sample GM-hb with the highest Pielou index (*J*_*e*_ = 0.63), it had the most uniform bacterial composition. The bacterial isolates were evenly distributed, with 50.00% *Bacillus*, 33.33% *Chryseobacterium,* and 16.67% *Pseudomonas*. Therefore, it can be concluded that the bacterial diversity of six soil samples in sequence from highest to lowest was KDG-f, GM-hb, BSC-f, GM-b, RB-c, and RB-hb (Figure 4, Table 4).

These results showed a significant difference in bacterial composition and diversity among the six soil samples, which indicated a possible relationship between soil properties and bacterial diversity. Liu et al. in 2020 [51] found that the variation in the composition of the microbial community was partly explained by the physiochemical properties of the soil, among which pH, total soil phosphorus, total nitrogen, and organic carbon were identified as dominant factors. In our future study, soil physiochemical properties should be analyzed to confirm the association with bacterial diversity. Moreover, recent studies have elucidated that soil physiochemical properties are regulated by various factors, such as altitude and cropping practices, which can cause variations in bacterial composition and diversity. Kumar et al. in 2019 [52] reported that Gangotri soil (altitude: 3,415 m) was richer in bacterial diversity than Kandakhal soil (1,532 m), as evidenced by a large number of different bacterial taxa and the higher Shannon–Wiener diversity index. This finding seems to partly explain our result that the highest altitude sample, BSC-f (3,480 m), had higher bacterial diversity than the lowest altitude soil sample, RB-hb (2,750 m), as evidenced by Shannon–Wiener index of 0.41 and 0.06, respectively.

More importantly, we found that forest soil samples without crop mulch (KDG-f and BSC-f) showed higher bacterial diversity compared to crop soil samples (GM-b, RB-c, and RB-hb). The result was attributed in part to the fact that cropping practices (tillage treatments) shaped the composition of the soil bacterial community, which was supported by previous studies. Liu et al. in 2020 [53] found that no-tillage treatment significantly increased the richness and uniformity of soil bacterial populations compared to conventional cereal and legume cropping practices. Furthermore, Yang et al. in 2020 [54] showed that the monoculture of different cover crops (millet, buckwheat, and bean) affected the compositions and distributions of the soil bacterial community, but did not differ significantly in bacterial richness and diversity. Their finding may partly contradict our result of a significant difference in bacterial diversity in crop soil samples. Therefore, it is of great value to provide deep insight into the correlation between cropping practices and soil bacterial diversity.

### 4.4. Determination of Amylase Activity from Bacterial Isolates

**Figure 5:**
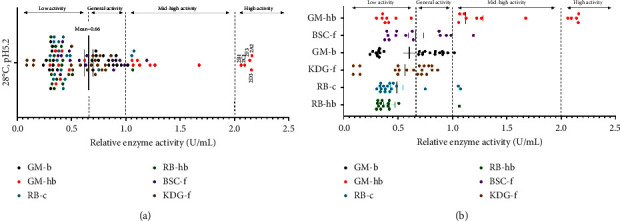
Distribution graph of amylase activity. (a) 110 bacterial isolates were intermingled together. The error bars represented the standard deviation of the mean for 110 amylase activities within three replicate values (*n* = 330). (b) The bacterial isolates were separated from six soil samples. Each error bar represented the standard deviation of the mean for each sample's amylase activity within three replicate values, GM-hb had 20 strains (*n* = 60), BSC-f had 14 strains (*n* = 42), GM-b had 25 strains (*n* = 75), KDG-f had 20 strains (*n* = 60), RB-c had 17 strains (*n* = 51), and RB-hb had 14 strains (*n* = 42).

Using the DNS method, the amylase activity assay was carried out to examine and compare the bacterial capacity for extracellular amylase production. As shown in Figure 5(a), the mean amylase activity of 110 bacterial isolates was 0.66 U/mL at 28°C, pH 5.2. According to the assay results, the levels of amylase activity were classified as follows: high activity (>2.00 U/mL), mid-high activity (1.00–2.00 U/mL); general activity (0.66–1.00 U/mL), and low activity (<0.66 U/mL). Therefore, 110 bacterial isolates were divided into four levels for amylase activity: 5 strains for high activity (4.55%), 12 strains for mid-high activity (10.91%), 28 strains for general activity (25.45%), and 65 strains for low activity (59.09%). In addition, the mean enzyme activity of six soil samples is shown in Figure 5(b), and the rank from high to low was GM-hb (1.12 U/mL), BSC-f (0.66 U/mL), GM-b (0.60 U/mL), KDG-f (0.56 U/mL), RB-c (0.49 U/mL), and RB-hb (0.42 U/mL).

Noticeably, five strains with high amylase activity all came from GM-hb (Figure 5(a)). The strains 2A2 (2.16 U/mL), 2D3 (2.16 U/mL), 2F3 (2.14 U/mL), 2C1 (2.09 U/mL), and 2B1 (2.06 U/mL) were identified as *Bacillus mycoides*, *Pseudomonas fluorescens*, *Chryseobacterium pennipullorum*, *C. pennipullorum*, and *C. jejuense*, respectively. What's more, 12 strains with mid high amylase activity also belonged to the above-mentioned genera: five strains to *Bacillus*, five strains to *Chryseobacterium,* and two strains to *Pseudomonas*. As a result, *Bacillus*, *Chryseobacterium*, and *Pseudomonas* seemed to be the major bacterial genera with relatively high amylase activity in our study.

### 4.5. Unique Enzyme Properties of Amylase-Producing Bacteria

#### 4.5.1. Effect of Temperatures and pHs on Amylase Activity

**Figure 6:**
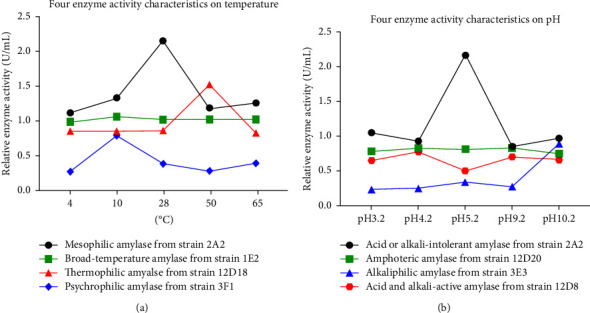
The effect of (a) temperatures and (b) pHs on amylase activity from representative strains.

Here, we further conducted the amylase activity assay under different temperatures (4, 10, 28, 50, and 65°C) and pHs (pH 3.2, 4.2, 5.2, 9.2, and 10.2) to determine how the factors affected the amylase activity of our bacterial isolates.  Figure 6 shows eight enzyme activity characteristics under the effects of temperatures and pHs.

On the one hand, four temperature characteristics were mesophilic amylase, broad temperature amylase, thermophilic amylase, and psychrophilic amylase, respectively (Figure 6(a)). Specifically, the mesophilic amylase showed an optimal temperature of 28°C, below or above which the activity was significantly reduced (*p value* <0.05). The decline in enzyme activity can be partially explained by the loss of the specific functional protein structure. These mesophilic amylases were produced from 6 strains (5.45%), 2A2, 2D3, 2F3, 2C1, 2B1, and 2B4, which belonged to the genera *Bacillus*, *Pseudomonas*, and *Chryseobacterium*. Broad temperature amylases activity did not show significant differences between 28°C and other temperatures, which were isolated from 102 strains (92.73%). Noticeably, a thermophilic amylase and a psychrophilic amylase were isolated from strains 12D18 and 3F1, respectively (Figure 6(a)). The thermophilic amylase from 12D18 displayed its optimum temperature of 50°C, at which enzyme activity increased from 0.86 U/mL (at 28°C) to 1.53 U/mL. The strain was identified as *Agrobacterium*, and other bacterial genera (*Bacillus*, *Proteus*, and *Pseudomonas*) were also reported for their maximum amylase activity at 50°C [47]. The thermal activity at 50°C is lower than previous reports for the optimal temperature of hyperthermophilic amylase from *Bacillus licheniformis* NH1 (90°C) [55] and *B. licheniformis* B4-423 (100°C) [11]. However, our thermophilic amylase with the highest activity at 50°C is likely to be favorable for use as a saccharifying enzyme in the starch process. As the starch liquefaction proceeds, a commixture of starch slurry and enzymes are first boiled to disintegrate the molecules and reduce the viscosity at 105–110°C, and then the partially disintegrated starch needs to be refrigerated to 50–60°C for further saccharifying [11]. Regarding the psychrophilic amylase, its highest activity of 0.79 U/mL was demonstrated at 10°C, and the producing bacterial isolate 3F1 belonged to *Bacillus mycoides*. It is well documented that *B. mycoides* is a psychrotolerant species, and some strains can grow at 7°C or below [56, 57], which may be one of the reasons why *B. mycoides* amylase exhibits the cold-active property. The cold environment may confer to the inhabited bacteria an adaptive characteristic to evolve a series of unique functional enzymes.

On the other hand, the effect of pH on amylase activity ranging from pH 3.2 to 10.2 showed four other enzyme characteristics: acid- or alkali-intolerant amylase, amphoteric amylase, alkaliphilic amylase, acid and alkali-active amylase (Figure 6(b)). Specifically, the acid- or alkali-intolerant amylase showed activity with a sharp decline below or above pH 5.2. Inactivity under extreme pH values may be attributed in part to denaturation of the enzyme structure. There were 41 strains (37.27%) isolated for acid, or alkali-intolerant amylase, including the six strains mentioned above with mesophilic amylase (2A2, 2D3, 2F3, 2C1, 2B1, and 2B4). While the amphoteric amylase exhibited its activity with no significant differences ranging from pH 3.2 to pH 10.2. A total of 42 strains (38.18%) showed their amylase activities within a wide pH range and were mostly identified as *Bacillus* (29 strains). Despite an earlier report on the amylase activity range of pH 3.5–7.0 from *Bacillus* sp. KR-8104 [19], our amphoteric amylases isolated from *Bacillus* appeared to have a broader activity range of pHs. Surprisingly, the alkaliphilic amylase from 24 strains (21.82%) displayed peak activity at pH 10.2. There are 17 strains isolated from sample RB-c, which triggered a hypothesis about whether the physiochemical property of the soil sample was so alkaline that the bacterial amylases worked best at an alkaline pH. Furthermore, our alkaliphilic amylases were isolated from *Bacillus* (15 strains), *Pseudomonas* (5 strains), *Chryseobacterium* (1 strain), *Klebsiella* (1 strain), *Rahnella* (1 strain), and *Raoultella* (1 strain), which can be concluded that the dominant bacterial genus for alkaliphilic amylase was *Bacillus*. The finding was consistent with the previous report that *Bacillus* sp. amylases have been used at an alkaline pH [58, 59]. A similar study found that the alkaline amylase from *Bacillus* sp. showed the maximum activity at pH 9.0 in the range of pH 8.0–10.0 [59], so in our study, the alkalophilicity at pH 10.2 is likely to be more prominent. Finally, the acid and alkali active amylase showed higher activity at pH 4.2 and 9.2, compared to the activity at pH 5.2. The producing bacterial isolates (12D7, 12D8, and 12D9) were identified as *Flavobacterium psychrophilum*, *Bacillus megaterium*, and *B. aryabhattai*, respectively. The acidophilic activity at pH 4.2 may confer an advantage to amylase in industrial applications. Currently, there is a great deal of interest in improving the low pH activity of industrial amylases. The starch liquefaction process is limited to being performed at pH 5.8–6.2, which is around the optimal pH of the present industrial amylases. Then, the postprocess steps such as saccharification need to be conducted at pH 4.3–4.8 [60, 61].

**Figure 7:**
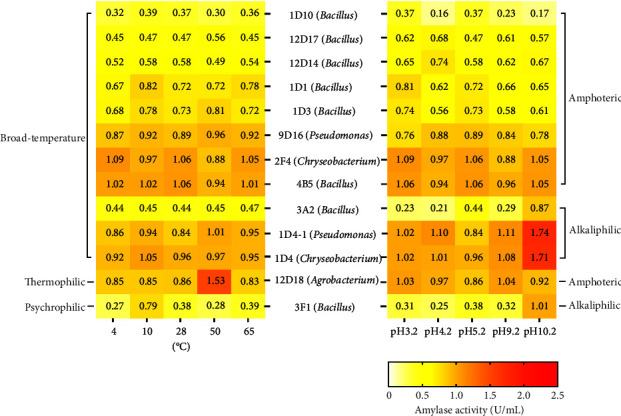
The representative bacterial isolates showed their dual characteristic of amylases on both temperature and pH activity. Each cell was labeled with the relative amylase activity (U/mL).

Finally, 13 representative bacterial isolates showed their dual characteristics of amylases on both temperature and pH activity, as can be seen in Figure 7. Isolates 1D10, 12D17, 12D14, 1D1, 1D3, 9D16, 2F4, and 4B5 can produce broad-temperature and amphoteric amylases. Strains 3A2, 1D4-1, and 1D4 were isolated for the broad-temperature and alkaliphilic amylases. Furthermore, there was a thermophilic and amphoteric amylase from strain 12D18 and a psychrophilic and alkaliphilic amylase from strain 3F1. Previous studies found that the genera *Proteus and Bacillus* were capable of producing thermophilic and alkaline amylase, with optimal activity at 50°C, pH 9.0 and 45°C, pH 8.5, respectively [47, 62]. However, the isolate 12D18 belonging to *Agrobacterium* exhibited the highest amylase activity at 50°C and pH 9.2 (Figure 7). Furthermore, the psychrophilic and alkaliphilic amylase from strain 3F1 (*Bacillus mycoides*) showed optimal activity at 10°C and pH 10.2 (Figure 7). Regarding the psychrophilic and alkaliphilic activities of amylase, our observation appears to be superior to a cold-active and alkaline amylase from *B. subtilis*, whose optimal activity was at 25°C and pH 8.0 [63].

#### 4.5.2. Bacterial Isolates for Multifunctional Enzyme Capacities

**Figure 8:**
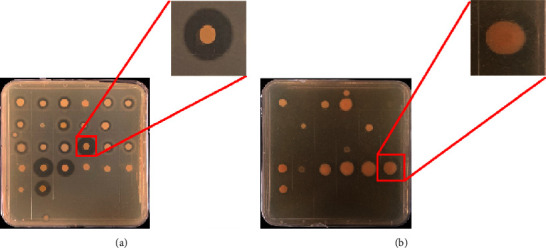
Amylase producing bacteria with (a) protease activity and (b) esterase activity.

**Figure 9:**
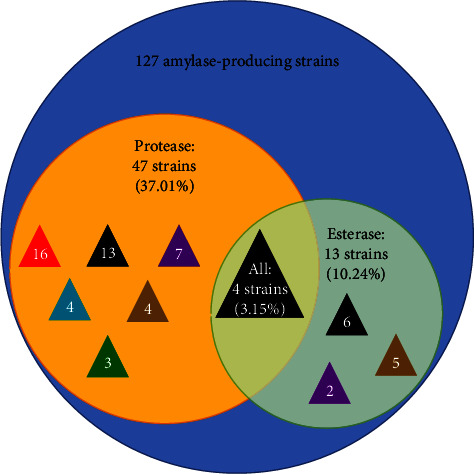
The number and percentage of 127 amylase producing bacteria isolates with protease and esterase activities. The triangle color indicated the sample source of strains (black: GM-b, red: GM-hb, blue: RB-c; green: RB-hb, purple: BSC-f, and brown: KDG-f).

We further explored whether the bacteria isolated for amylase had other functional enzyme activities by the method of an activity-based screening (Figure 8). There were 47 strains (37.01%) producing amylase and protease activities, which were mainly identified as *Bacillus* (32 strains), followed by *Chryseobacterium* (8 strains), *Exiguobacterium* (3 strains), *Pseudomonas* (2 strains), *Microbacterium* (1 strain), and *Paenibacillus* (1 strain). 13 strains (10.24%) were capable of amylase and esterase activities, belonging to three bacterial genera: *Bacillus* (6 strains), *Pseudomonas* (6 strains), and *Flavobacterium* (1 strain). Noticeably, there were four bacterial isolates (3.15%) isolated for amylase, protease, and esterase, as shown in Figure 9. The four strains for the triple-functional enzyme activities were identified as *Bacillus aryabhattai* (1D1, 1D3, and 1D11) and *Pseudomonas fluorescens* (1E2). According to the above-mentioned study on amylase characteristics, strains 1D1 and 1D3 were isolated for the broad-temperature and amphoteric amylases (Figure 7). These bacterial isolates are likely to get more attention for scientific studies and industrial applications.

## 5. Conclusions

Tibetan soil is likely to provide a unique environment conducive to inhabited bacteria to produce functional enzymes with superior activities. In this study, 127 amylase-producing bacteria were isolated from 6 soil samples collected in Nyingchi, Tibet. Among the 12 identified bacterial genera, *Bacillus* was supposed to be the dominant genera for amylase activity. Regarding the diversity of the bacterial community, we find variation appearing in the soil microbial, which may be partly explained by the soil altitude and cropping practice. Moreover, we obtained a psychrophilic and alkaliphilic amylase from the bacterial isolate 3F1 (*Bacillus mycoides*). Of course, further studies are required to reveal the critical amino acid residues (structural determinants) for cold resistance and alkaline-adaption. Consequently, the bacterial isolates in this study are likely to have potential to be further utilized for the amylase industry.

## Figures and Tables

**Table 1:** Information on six soil samples.

Sample	Sampling position	Longitude (E)	Latitude (N)	Altitude (m)	Soil type
GM-b	Guomo village, Mainling County	94° 42′	29° 26′	3,033	Buckwheat
GM-hb	Guomo village, Mainling County	94° 42′	29° 26′	3,033	Highland barley
RB-c	Runa village, Bomê County	95° 36′	30° 00′	2,750	Corn
RB-hb	Runa village, Bomê County	95 36′	30° 00′	2,750	Highland barley
BSC-f	Basongcuo	93 54′	30 00′	3,480	Forest
KDG-f	Kadinggou	94° 10′	29° 45′	2,980	Forest

**Table 2:** Isolation and screening of amylase-producing bacteria from six Tibetan soil samples.

Sample	Average bacterial colony counts	Number of amylase-producingstrains	Isolation proportion (%)
GM-b	174	27	15.52
GM-hb	292	24	8.22
RB-c	248	24	9.68
RB-hb	213	16	7.51
BSC-f	137	15	10.95
KDG-f	120	21	17.50

**Table 3:** BLAST results of the redundant strains based on 16S rDNA sequence similarity.

Bacteria ID	Closest representative bacteria	Sequence similarity (%)
1D2 (1D21)	*Bacillus* mycoides BF1-5	100.00
4D5 (4G5)	*Bacillus mycoides* DSM 2048	100.00
9D7 (9D11)	*Bacillus mycoides* BPN401	100.00
3C1 (3E1, 3C3, 3F3)	*Bacillus mycoides* TS26	100.00
3F2 (3G3, 3H3)	*Bacillus mycoides* TS27	100.00
1D8 (1D22)	*Bacillus aryabhattai* PF4_AM	100.00
3E3 (3H1)	*Bacillus toyonensis* FORT 102	100.00
2C2 (2C5, 2H5)	*Bacillus mycoides* DSM 2048	99.93
2A6 (2G4, 2D5)	*Pseudomonas fluorescens *Pf0-1	99.93
12D1 (12D15)	*Flavobacterium glaciei* EA2-2	99.93
3D2 (3E2)	*Raoultella terrigena* JCM 1687	99.93
4B5 (4C6)	*Bacillus mycoides* T1-18	99.86

Notes. The redundant strains were in parentheses.

**Table 4:** Bacterial diversity index of six soil samples.

Sample	Berger–Parker index (*D*_*B-P*_)	Margalef index (*d*_*Ma*_)	Simpson index (*D*)	Shannon–Wiener index (*H′*)	Pielou index (*J*_*e*_)
KDG-f	0.43	1.97	0.69	0.85	0.44
GM-hb	0.50	0.63	0.61	0.69	0.63
BSC-f	0.67	1.48	0.52	0.41	0.25
GM-b	0.74	0.61	0.40	0.30	0.27
RB-c	0.83	0.94	0.30	0.18	0.13
RB-hb	0.94	0.36	0.12	0.06	0.09

## Data Availability

Excel and other data files used to support the findings of this study are available upon request from the corresponding author.
